# 1029 genomes of self-declared healthy individuals from India reveal prevalent and clinically relevant cardiac ion channelopathy variants

**DOI:** 10.1186/s40246-022-00402-2

**Published:** 2022-08-05

**Authors:** Anjali Bajaj, Vigneshwar Senthivel, Rahul Bhoyar, Abhinav Jain, Mohamed Imran, Mercy Rophina, Mohit Kumar Divakar, Bani Jolly, Ankit Verma, Anushree Mishra, Disha Sharma, Siddharthan Deepti, Gautam Sharma, Raghav Bansal, Rakesh Yadav, Vinod Scaria, Nitish Naik, Sridhar Sivasubbu

**Affiliations:** 1 CSIR-Institute of Genomics and Integrative Biology, Sukhdev Vihar, Mathura Road, New Delhi, 110025 India; 2 Academy of Scientific and Innovative Research (AcSIR), Ghaziabad, 201002 India; 3 Department of Cardiology, All India Institute of Medical Sciences (AIIMS), New Delhi, 110029 India

**Keywords:** Cardiac channelopathies, IndiGen, Genome sequencing, Prevalence, Indian population, ACMG/AMP, Population screening

## Abstract

**Background:**

The prevalence and genetic spectrum of cardiac channelopathies exhibit population-specific differences. We aimed to understand the spectrum of cardiac channelopathy-associated variations in India, which is characterised by a genetically diverse population and is largely understudied in the context of these disorders.

**Results:**

We utilised the IndiGenomes dataset comprising 1029 whole genomes from self-declared healthy individuals as a template to filter variants in 36 genes known to cause cardiac channelopathies. Our analysis revealed 186,782 variants, of which we filtered 470 variants that were identified as possibly pathogenic (440 nonsynonymous, 30 high-confidence predicted loss of function ). About 26% (124 out of 470) of these variants were unique to the Indian population as they were not reported in the global population datasets and published literature. Classification of 470 variants by ACMG/AMP guidelines unveiled 13 pathogenic/likely pathogenic (P/LP) variants mapping to 19 out of the 1029 individuals. Further query of 53 probands in an independent cohort of cardiac channelopathy, using exome sequencing, revealed the presence of 3 out of the 13 P/LP variants. The identification of p.G179Sfs*62, p.R823W and c.420 + 2 T > C variants in *KCNQ1, KCNH2* and *CASQ2* genes, respectively, validate the significance of the P/LP variants in the context of clinical applicability as well as for large-scale population analysis.

**Conclusion:**

A compendium of ACMG/AMP classified cardiac channelopathy variants in 1029 self-declared healthy Indian population was created. A conservative genotypic prevalence was estimated to be 0.9–1.8% which poses a huge public health burden for a country with large population size like India. In the majority of cases, these disorders are manageable and the risk of sudden cardiac death can be alleviated by appropriate lifestyle modifications as well as treatment regimens/clinical interventions. Clinical utility of the obtained variants was demonstrated using a cardiac channelopathy patient cohort. Our study emphasises the need for large-scale population screening to identify at-risk individuals and take preventive measures. However, we suggest cautious clinical interpretation to be exercised by taking other cardiac channelopathy risk factors into account.

**Supplementary Information:**

The online version contains supplementary material available at 10.1186/s40246-022-00402-2.

## Background

Inherited cardiac ion channelopathies are a group of rare genetic disorders characterised by electrical disturbances in the heart [1]. These disorders primarily include Long QT Syndrome (LQTS), Brugada Syndrome (BrS), Catecholaminergic Polymorphic Ventricular Tachycardia (CPVT) and Short QT Syndrome (SQTS). Approximately 10–30% of sudden unexpected deaths in young adults (< 35 years) with a negative autopsy for structural heart disease have been suspected to be attributable to cardiac ion channelopathies [2–4]. Keating and colleagues, for the first time, identified the genetic basis underlying channelopathy disorders and discovered three key genes namely, *KCNH2*, *SCN5A* and *KCNQ1* [5–7]. So far, over 36 genes have been associated with cardiac ion channelopathies [8]. About 15,000 variations in these 36 genes have been identified to be associated with cardiovascular phenotypes [9]. These genes code for the ion channels and their accessory subunits in the cardiac muscle cells. There is a degree of overlap in the involvement of different ion channels to cause the same syndrome and *vice versa*. It is well established that genotypic and phenotypic heterogeneity is a hallmark of these disorders [8]. In the majority of cases, different types of channelopathies can be differentiated by distinct ECG wave patterns. However, genetic testing provides an immense aid in confirmatory diagnosis as well as the screening and assessment of at-risk family members.

Advancements in the next-generation sequencing technologies have considerably decreased the cost of sequencing and increased its throughput. With the availability of global population genomic datasets such as the 1000 genomes project [10], gnomAD [11] and ESP6500 [12], it is now possible to estimate the allele frequency of the rare variations in the general population. This aids in evaluating the prevalence of previously reported disease-associated variations and investigating their clinical actionability. For instance, a set of 33 variants previously reported to cause LQTS were found to affect 173 alleles in the ESP dataset which comprises 5400 individuals [13]. Based on this, the genotype prevalence of LQTS was deduced to be 1:31 in the ESP population. Moreover, genomic initiatives may bring out ethnic differences in the disease-associated variations and susceptibilities. Kong and colleagues reported meta-analysis of the ion channelopathy genes across different ethnicities and revealed that the allele frequency distribution was significantly different amongst different groups with the Asians carrying the most alleles in sudden cardiac death-associated genes [14].

Amongst Asians, the Indian population is culturally heterogeneous with 4000 anthropologically distinct groups speaking more than 300 languages. The practice of specific marriage patterns has led to the formation of multiple endogamous groups and enrichment of deleterious recessive alleles [15]. These population features and strict practices have made the Indian population genetically diverse and distinct from the rest of the world. Owing to these features, there is an immense opportunity to discover the unique genetic spectrum of cardiac ion channelopathy variants in this subcontinent. In context of several other diseases and pharmacogenetics, it is well established that there exists population-specific variants or sub-population-specific unique variants [16–18]. Due to this, the knowledge about genetic variations and their frequency in global populations are not completely aligned with the Indian population. Thus, it is important to document the disease-associated genotypes and their prevalence in Indian settings. However, with the exception of a handful of genome-scale studies [19, 20], population-specific high-throughput genomic studies have been scarce in India. Moreover, there have been very few studies that elucidate the genotype–phenotype correlation in cardiac channelopathies in India. These two factors have posed a barrier in understanding the prevalence and clinical implications of cardiac channelopathy-associated rare variants in this part of the world. The aim of creating an Indian population-specific genomic dataset propelled the completion of pilot phase of the IndiGen study in which whole genomes of 1029 self-declared healthy individuals representing different ethnic groups across India were sequenced [21, 22]. The IndiGenomes dataset serves as one of the starting points for deriving Indian population-specific genotypic prevalence estimates in context of various rare genetic disease conditions.

In this study, we utilised the IndiGenomes dataset to discover the genetic spectrum underlying cardiac channelopathies in self-declared healthy Indian individuals. Analysis of 1029 personal genomes revealed 440 nonsynonymous and 30 high confidence pLoF variants. Although copy number variations and other structural genomic variants can also be important genetic players for cardiac channelopathies, we limited our analysis to nonsynonymous variants and loss of function variants. We observed that 26% of the variants were exclusive to the Indian population. Further classification of the variants using ACMG/AMP guidelines revealed 13 P/LP variants mapping to 19 individuals or conservatively 9 P/LP variants mapping to 10 individuals, which translates to about 1 in 54 individuals or 1 in 103 individuals, respectively, harbouring a variant(s) that may cause cardiac channelopathies. This dataset was further validated on a patient cohort underscoring the importance of the study and indicating a large at-risk population in the Indian subcontinent.

## Materials and methods

### IndiGen study population

A total of 1029 self-declared healthy individuals, with their ancestries mapping to different geographical locations spread across India, consensually volunteered for the IndiGen study. The variants in genome sequence data and their allele frequencies were previously published by our group through the IndiGenomes database [21].

### Data generation and analysis

Both IndiGenomes and patient cohort dataset were generated on Illumina platforms using sequencing by synthesis chemistry. The IndiGenomes dataset comprises whole-genome sequences and the patient cohort data consists of whole-exome sequences. There were following differences in the data generation as well as analysis pipelines.

#### Data generation

IndiGen WGS: The sequencing libraries were prepared from fragmented DNA using the TruSeq DNA PCR free LT sample preparation kit as instructed by the manufacturer (Illumina Inc.). The sequencing-ready libraries were further processed for paired-end sequencing on Illumina NovaSeq6000 (S4 flowcell) instrument.

Patient cohort WES: Whole-exome sequencing libraries were prepared from the fragmented DNA samples using Illumina TruSeq DNA or Nextera exome kit as instructed by the manufacturer (Illumina Inc.). The pooled libraries were sequenced on the Illumina sequencing platform (HiSeq2500 and NovaSeq6000) generating paired-end reads.

The IndiGenomes dataset was generated exclusively on Novaseq6000 platform in 6 months whereas the exome sequencing dataset from the patient cohort was generated over a time period of 5 years using both HiSeq2500 and NovaSeq6000 platforms.

#### Data analysis

IndiGen WGS: Alignment, post-processing and default quality filtered variant calling was performed on the Illumina DRAGEN v3.4 Bio-IT platform (Illumina Inc. San Diego, CA, USA) using GRCh38 as a human reference genome. The joint variant calling was performed using Sentieon. The variants were systematically annotated using ANNOVAR.

Patient cohort WES: Alignment, post-processing and default quality filtered variant calling was performed on the Illumina DRAGEN v3.4 Bio-IT platform (Illumina Inc. San Diego, CA, USA) using GRCh38 as a human reference genome. The variants were systematically annotated using ANNOVAR.

### Filtering Indigenomes dataset

The variant positions were based on the build GRCh38/hg38 of human genome assembly and were annotated using ANNOVAR (v.2018–04-16) [23], a software tool integrating multiple databases and computational tools for functional annotation. In summary, it comprises annotations from RefGene [24], dbSNP [25] and dbNSFP35a [26], and various global population databases such as Genome Aggregation Database (gnomAD) [11], 1000 Genome project [10], NHLBI GO Exome Sequencing Project (ESP6500) [12], Exome Aggregation Consortium (ExAC) [27] and Greater Middle East (GME) Variome Project [28]. For functional effect interpretation, dbNSFP35a [26] database was used. It includes various variant pathogenicity prediction tools such as SIFT, PolyPhen, LRT, MutationTaster, PROVEAN, MutationAccessor, FATHMM, RadialSVM, CADD, DANN and phyloP amongst others. In order to assess the clinical significance of the variants, ClinVar [9] (hg38_clinvar_20210501) was used.

**Fig. 1:**
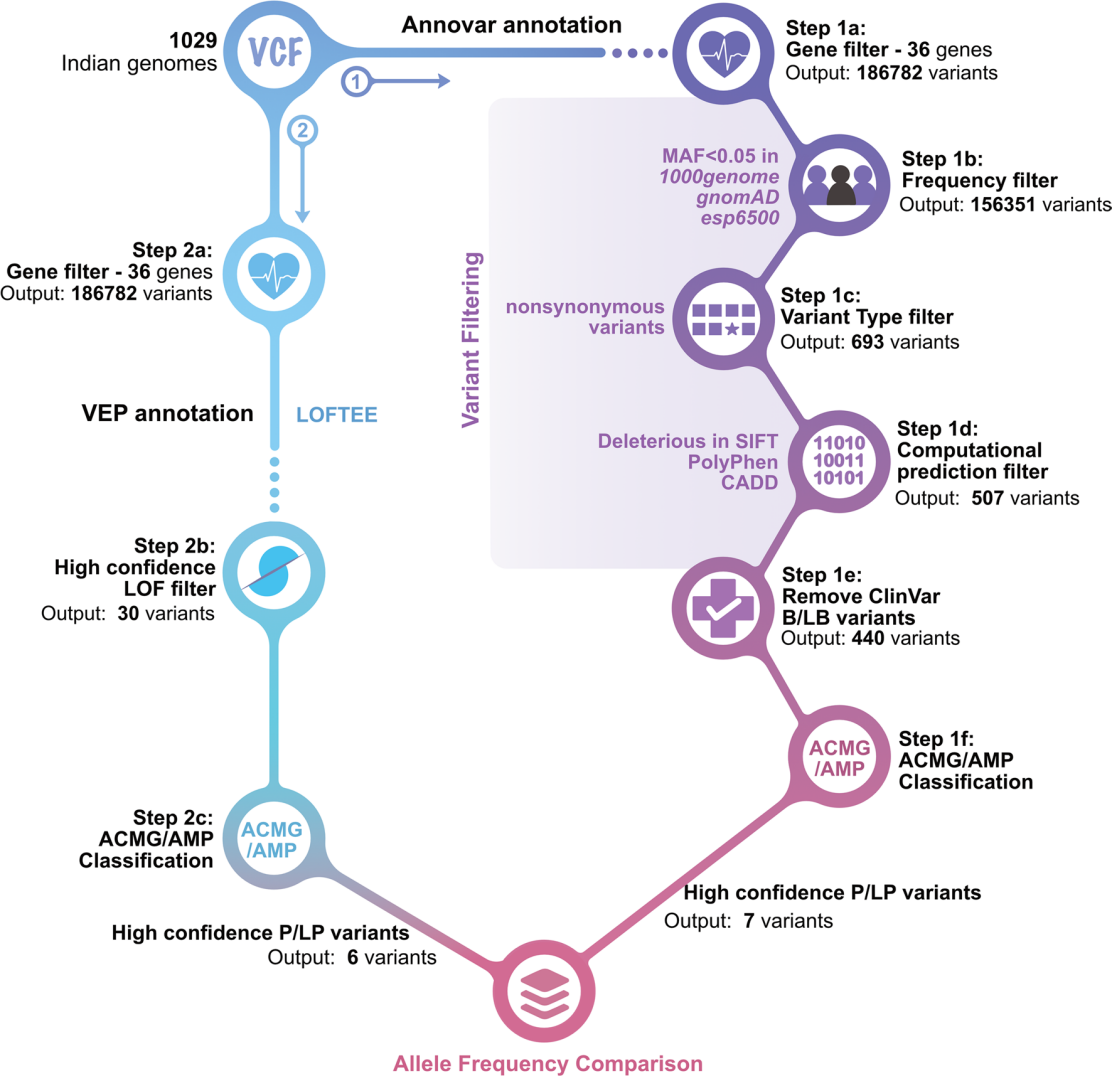
Schematic of the workflow for variant filtering strategy and the allele frequency comparisons: (1) ANNOVAR annotation for filtering nonsynonymous variants (2) VEP annotation for pLoF variants, B/LB—Benign/likely benign, P/LP—Pathogenic/likely pathogenic, ACMG/AMP: American College of Medical Genetics and Genomics/ Association for Molecular Pathology, LOF—predicted loss of function

For our analysis, we selected a list of 36 genes associated with cardiac ion channelopathy disorders. A subset of 12 genes has strong/ definitive evidence for pathogenicity. On the other hand, the remaining 24 genes are of disputed/limited evidence for inherited form of the disease [29, 30]. Despite being categorised as limited/disputed evidence, *KCNE1* and *KCNE2* have strong evidence for acquired LQTS (Additional file 1). The variants falling within the shortlisted 36 cardiac channelopathy genes (Additional file 1) were filtered and subjected to further analysis (Fig. 1). To obtain the rare variants, an allele frequency cut-off of MAF < 0.05 was applied in population datasets that were included in the ANNOVAR tool namely, 1000 Genomes, gnomAD and ESP6500 and the resulting rare variants were further filtered for only exonic and nonsynonymous variants. A filter was applied in which all the variants predicted to be deleterious by at least one of the three tools, i.e. SIFT (D), PolyPhen (D or P), CADD (score > 15), were selected. A final filter to remove all the benign variants reported in ClinVar was applied. The resulting list of variants was taken further for classification using ACMG/AMP guidelines.

### Annotation of predicted loss of function (pLoF) variants

From the genome sequencing data of 1029 individuals, all pLoF variants present in the 36 shortlisted cardiac channelopathy genes were annotated using Ensembl—Variant effect Predictor tool (VEP—version 98) [31] with loss of function transcript effect estimator (LOFTEE) [11] plug-in (Fig. 1). The function of this plug-in module is to annotate all frameshift, stopgain and splice-site variants and classify them as high confidence or low confidence predicted loss of function (pLoF) variants.

### ACMG/AMP classification

Nonsynonymous as well as pLoF variants were individually assessed and classified according to the ACMG/AMP guidelines [32]. In order to reduce the disparity between annotations for nonsynonymous (ANNOVAR package) and pLoF (LOFTEE package) variants, we have considered Matched Annotation from the NCBI and EMBL-EBI (MANE) transcripts wherever it was possible. Attributes were assigned to each variant based on the consensus between two curators. The detailed criteria have been explained in Additional file 2. After the attributes were assigned, classification was done using the Genetic Variant Interpretation Tool [33].

### Unique cardiac channelopathy variants filtering

For obtaining unique variants in the IndiGenomes dataset, we compared the variants that were obtained after variant filtering (Fig. 1) with major population datasets (1000genome, gnomAD, ExAC, ESP6500, GME), publicly available databases (ClinVar, dbSNP) and published literature. If a variant was absent in all of these resources and was only present in the IndiGenomes dataset, then it was considered a unique variant for the Indian population.

### Allele frequency comparisons

Allele frequency of all the variants in the IndiGenomes dataset was previously calculated [21]. The frequencies of P/LP variants were compared with the existing population databases such as the 1000 Genomes project, Genome Aggregation Database (gnomAD v3.1.2 and gnomAD v2.1.1), ESP6500 (esp6500siv2_all), GME, Qatar [34] and GenomeAsia100K. Additionally, SAS sub-population datasets were also used. In order to ascertain significant differences in the allele frequencies of IndiGenomes dataset, Fisher’s exact test was performed.

### Validation of P/LP variations in exome sequence dataset of independent patient cohort

As part of the Genomics for Understanding Rare Diseases: India Alliance Network (GUaRDIAN) [35] consortium, a cohort of patients (*n* = 53) with a provisional diagnosis of cardiac channelopathy disorders was established in collaboration with the All India Institute of Medical Sciences (AIIMS), a tertiary healthcare centre in India. The genomic characterisation of the probands was done by exome sequencing and the presence of P/LP variations obtained from the IndiGenomes study was checked in this cohort.

## Results

### Analysis of variants in cardiac ion channelopathy-associated genes from IndiGenomes dataset

The IndiGenomes dataset comprises about 56 million genetic variants from the genomes of 1029 self-declared healthy Indian individuals. Out of these, over 18 million genetic variants are unique to the Indian population [21]. We used this dataset and extracted a total of 186,782 variants present in 36 cardiac ion channelopathy-associated genes (Additional file 1).

For analysis of nonsynonymous variations, ANNOVAR annotations on the IndiGenomes dataset were used [21]. By applying a variant filtering pipeline (Fig. 1), we analysed the spectrum of rare and probably pathogenic variants associated with cardiac ion channelopathies. Firstly, by applying a conservative cut-off of MAF < 0.05 in major population datasets, we obtained a compendium of 156,351 rare variants. Out of these, 1263 were exonic variants. Their mapping with respect to the reference gene annotations has been summarised in Additional file 3. Next, we retrieved all the exonic nonsynonymous SNVs which accounted for a total of 693 variants. To obtain the probably pathogenic variants, we selected predicted deleterious variants as annotated from SIFT, PolyPhen or CADD and removed benign or likely benign variants reported in the ClinVar database. As a result, we were left with a corpus of 440 nonsynonymous variants.

### Interpretation of nonsynonymous variants according to ACMG/AMP guidelines

A total of 440 nonsynonymous exonic variants in 36 genes were obtained after applying the variant filtering pipeline. These were taken ahead for in-depth genetic interpretation based on the ACMG/AMP guidelines [32] and the final classification was carried out using Genetic Variant Interpretation Tool [33].

**Fig. 2:**
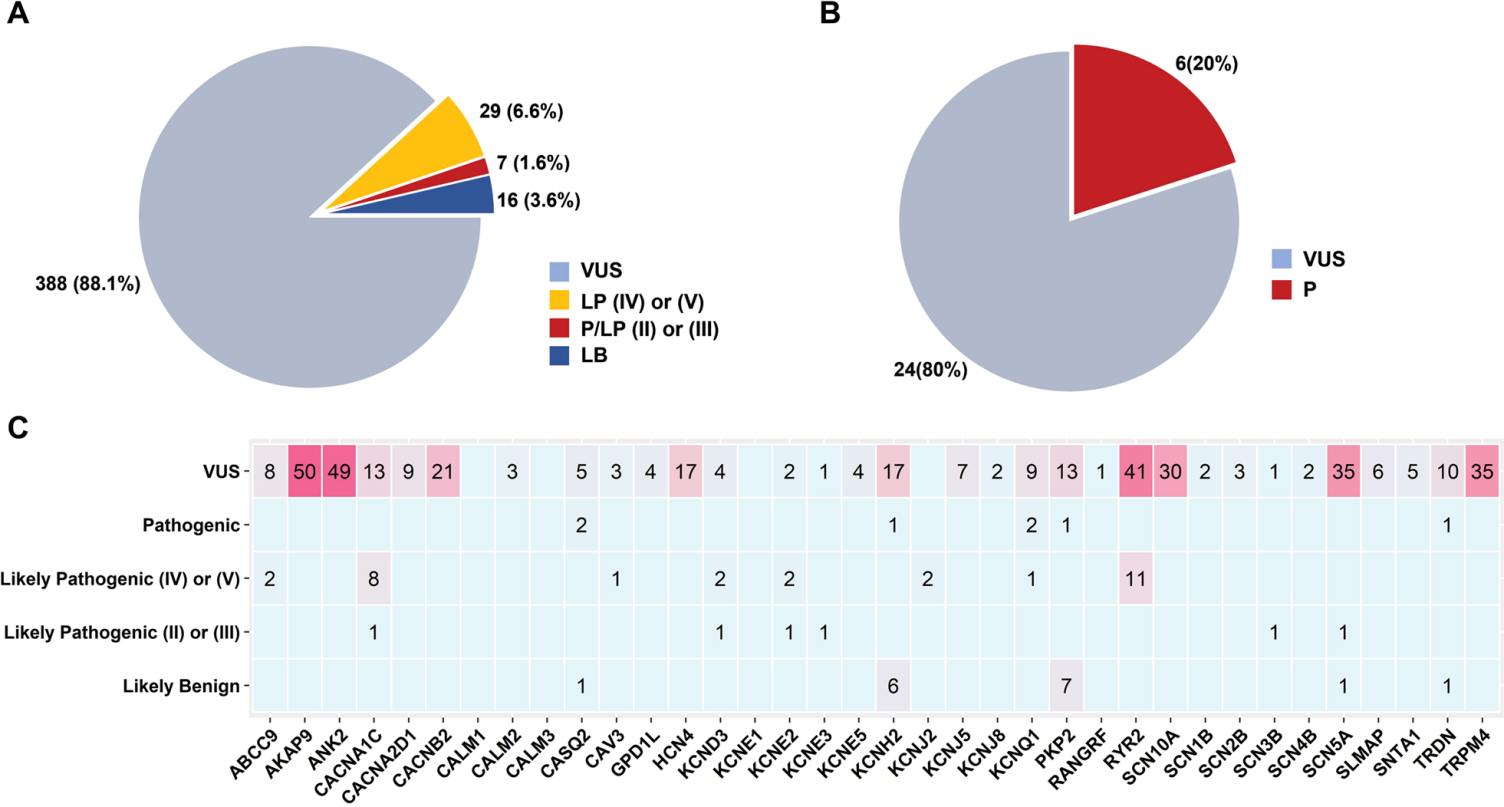
Distribution of ACMG/AMP classified variants associated with cardiac ion channelopathy.** A** Pie chart representing the percentage of nonsynonymous variants classified as VUS, LP(IV) or (V), P/LP(II) or (III) and LB. **B** Pie chart representing the percentage of pLoF variants classified as VUS and P. **C** Heat map representing the distribution of 470 ACMG/AMP classified variants (nonsynonymous and pLoF) across 36 cardiac ion channelopathy genes. Red colour gradient corresponds to the number of variants in a gene. Numbers in rectangles represent the number of variants

A subset of 36 variants was classified as pathogenic (*n* = 1) or likely pathogenic (*n* = 35). Depending on the available strength of evidence, particularly in the context of functional studies, the variants in the likely pathogenic category were divided into high confidence (likely pathogenic (II/III), *n* = 6) and low confidence (likely pathogenic (IV/V), *n* = 29) (Fig. 2A). Moreover, 16 variations were classified as likely benign, out of which, 13 variations were present in *KCNH2* (*n* = 6) and *PKP2* (*n* = 7) genes. A majority of variants (*n* = 388) were classified as variants of uncertain significance (VUS). This category was assigned in cases where there was a lack of evidence or conflicting evidence for interpreting the pathogenicity of the variant. Of all the variants classified as VUS, more than half (58.2%) were harboured in 6 genes: *AKAP9, ANK2, RYR2, SCN10A, SCN5A* and *TRPM4* (Fig. 2C).

In total, we have identified 7 pathogenic or high confidence likely pathogenic nonsynonymous variants. These variants were found to be present in 12 out of the 1029 individuals analysed. The variants *KCNH2*:p.R823W, *SCN5A*:p.V2016M and *CACNA1C*:p.R858H were present in 1 individual each whereas the variants *KCNE2*:p.I57T, *KCND3*:p.L450F and *KCNE3*:p.V17M were present in 2 individuals each. The variant *SCN3B*:p.V110I was present in 3 individuals. All of the 12 individuals carry the respective variants in heterozygous state. Additionally, all these 7 variants have been independently validated by sanger capillary sequencing (primer details mentioned in Additional file 4). Considering that the disorders associated with the above-mentioned genes follow an autosomal dominant mode of inheritance, individuals carrying the variants in these genes may be at risk of developing a channelopathy disorder.

The details of all 36 P/LP variants are summarised in Table 1. The complete list of 440 nonsynonymous variations with their ACMG/AMP annotations is elaborated in Additional file 5.

**Table 1:** Pathogenic and likely pathogenic cardiac channelopathy variants as per ACMG/AMP guidelines

Gene	cDNA change	AA change	dbSNP ID	Functional or segregation evidence	ClinVar	ACMG/AMP attributes	ACMG/AMP classification
KCND3	c.1348C > T	p.L450F	rs150401343	Gain of function of KV4.3/KChIP2‑encoded channels (PMID: 26016905)	Conflicting_interpretations_of_pathogenicity	PS3PM2PP2BP4	Likely Pathogenic(II)
SCN5A	c.6046G > A	p.V2016M	rs762981322	Loss of function by reduced cell surface expression and peak Na+ current (PMID: 24895455 and PMID: 26282245) and gain of function by protein kinase A or C activation (PMID: 26282245)	Conflicting_interpretations_of_pathogenicity	PS3PM2PP3	Likely Pathogenic(II)
KCNH2	c.2467C > T	p.R823W	rs199473538	Trafficking defect (PMID: 16432067) and Failure to return to normal repolarisation (PMID: 23303164)	Pathogenic/likely_pathogenic	PS3PM1PM2PP3PP5BP1	Pathogenic(IIIb)
KCNE3	c.49G > A	p.V17M	rs773287275	Gain of function on Kv4.3/KCNE3 and Kv11.1/KCNE3 generated currents (PMID: 18209471)	Uncertain_significance	PS3PM2PP3	Likely Pathogenic(II)
SCN3B	c.328G > A	p.V110I	rs147205617	Loss of function by reducing sodium current (23257389)	Conflicting_interpretations_of_pathogenicity	PS3PM1PP3	Likely Pathogenic(II)
CACNA1C	c.2573G > A	p.R858H	rs786205753	Gain of function on Ica (PMID: 24728418)	Pathogenic/likely_pathogenic	PS3PM2PP2PP3PP5	Likely Pathogenic(II)
KCNE2	c.170T > C	p.I57T	rs74315448	Gain of function of Ito (PMID: 20042375), loss of function of Iks (PMID: 11101505), loss of function of Ikr (PMID: 10219239)	Conflicting_interpretations_of_pathogenicity	PS3PM1PP2PP3	Likely Pathogenic(II)
KCND3	c.1649G > A	p.R550H	rs151164490	NA	Uncertain_significance	PM1PM2PP2PP3	Likely Pathogenic(V)
KCND3	c.325G > C^$^	p.E109Q	NA	NA	NA	PM1PM2PP2PP3	Likely Pathogenic(V)
RYR2	c.356 T > C^$^	p.I119T	NA	NA	NA	PM1PM2PP2PP3	Likely Pathogenic(V)
RYR2	c.745 T > C^$^	p.S249P	NA	NA	NA	PM1PM2PP2PP3	Likely Pathogenic(V)
RYR2	c.892C > T	p.R298C	rs551099887	NA	Conflicting_interpretations_of_pathogenicity	PM1PM2PP2PP3	Likely Pathogenic(V)
RYR2	c.1396C > T^$^	p.P466S	NA	NA	NA	PM1PM2PP2PP3	Likely Pathogenic(V)
RYR2	c.1910 T > G^$^	p.L637R	NA	NA	NA	PM1PM2PP2PP3	Likely Pathogenic(V)
RYR2	c.1940G > A	p.R647H	rs766330093	NA	Uncertain_significance	PM1PM2PP2PP3	Likely Pathogenic(V)
RYR2	c.4388G > A	p.R1463H	rs753707154	NA	NA	PM1PM2PP2PP3	Likely Pathogenic(V)
RYR2	c.4531G > T	p.V1511L	NA	NA	Uncertain_significance	PM1PM2PP2PP3	Likely Pathogenic(V)
RYR2	c.6367C > T	p.L2123F	rs1060500159	NA	Uncertain_significance	PM1PM2PP2PP3	Likely Pathogenic(V)
RYR2	c.11758C > A^$^	p.L3920I	NA	NA	NA	PM1PM2PP2PP3	Likely Pathogenic(V)
RYR2	c.13526 T > C	p.V4509A	NA	NA	Uncertain_significance	PM1PM2PP2PP3	Likely Pathogenic(V)
CAV3	c.244G > T^$^	p.V82F	NA	NA	NA	PM1PM2PP2PP3	Likely Pathogenic(V)
KCNQ1	c.1570G > T^$^	p.V524L	NA	NA	NA	PM1PM2PM5BP4	Likely Pathogenic(IV)
CACNA1C	c.1914C > A	p.S638R	NA	NA	NA	PM1PM2PP2PP3	Likely Pathogenic(V)
CACNA1C	c.2813 T > C	p.I938T	rs377165829	NA	Uncertain_significance	PM1PM2PP2PP3	Likely Pathogenic(V)
CACNA1C	c.2837 T > C	p.I946T	rs747728381	NA	Uncertain_significance	PM1PM2PP2PP3	Likely Pathogenic(V)
CACNA1C	c.3200C > T	p.A1067V	rs750998195	NA	Conflicting_interpretations_of_pathogenicity	PM1PM2PP2PP3	Likely Pathogenic(V)
CACNA1C	c.3691C > T	p.L1231F	rs766971426	NA	NA	PM1PM2PP2PP3	Likely Pathogenic(V)
CACNA1C	c.4819C > T	p.P1607S	rs745938574	NA	Uncertain_significance	PM1PM2PP2PP3	Likely Pathogenic(V)
CACNA1C	c.5270G > T	p.S1757I	rs753388569	NA	NA	PM1PM2PP2PP3	Likely Pathogenic(V)
CACNA1C	c.5684G > A	p.R1895Q	rs753954220	NA	Uncertain_significance	PM2PP2PP3PM1	Likely Pathogenic(V)
ABCC9	c.2474C > T	p.A825V	rs964127282	NA	Uncertain_significance	PM1PM2PP2PP3	Likely Pathogenic(V)
ABCC9	c.1318C > G^$^	p.Q440E	NA	NA	NA	PM1PM2PP2PP3	Likely Pathogenic(V)
KCNJ2	c.208G > C^$^	p.A70P	NA	NA	NA	PM1PM2PP2PP3	Likely Pathogenic(V)
KCNJ2	c.817A > G^$^	p.I273V	NA	NA	NA	PM1PM2PP2PP3	Likely Pathogenic(V)
KCNE2	c.131A > G^$^	p.E44G	NA	NA	NA	PM1PM2PP2PP3	Likely Pathogenic(V)
KCNE2	c.229C > T	p.R77W	rs141423405	No effect on Ikr (PMID: 17275752)	Uncertain_significance	PM1PM2PP2PP3BS3	Likely Pathogenic(V)

*AA* amino acid; *Ica* calcium current; *Ikr* rapid delayed rectifier potassium current; *Iks* slow delayed rectifier potassium current; *NA* not available; *ACMG/AMP* American College of Medical Genetics and Genomics/Association for Molecular Pathology; *PMID *pubmed id for functional study reference; *$* unique variants

Owing to incomplete penetrance of the disease, strong evidence to ascertain pathogenicity in the context of segregation studies was lacking in the literature for almost all of the variations. Thus, functional studies became the major determinant. For instance, a p.R823W variation in the *KCNH2* gene was classified as a pathogenic variant as it has been demonstrated to cause trafficking defects by in vitro study [36] and loss of function phenotype using zebrafish [37] as well as high-throughput electrophysiological phenotyping [38]. Another variation, p.V2016M in the *SCN5A* gene was classified as high confidence likely pathogenic since it had been shown to reduce cell surface expression and peak Na + currents in HEK293 cells [39]. Moreover, mice experiments in the same study have shown that the SIV domain spanning the amino acid valine plays an important role in the correct expression of Nav1.5 in the lateral myocyte membrane, which is further important for cardiac conduction. Another study for the p.V2016M variation reported that it exhibits loss of function as well as gain of function features by protein kinase A activation or C activation [40].

### Interpretation of predicted loss of function variants

Loss of function variants include splicing, stopgain and frameshift variants. They can have deleterious effects on the protein function and thus, can potentially cause the disease. We evaluated pLoF variants separately in the IndiGenomes dataset which mapped to the 36 cardiac ion channelopathy genes. Variants were annotated from Variant effect Predictor tool (VEP) and using loss of function transcript effect estimator (LOFTEE), we predicted 30 high confidence LoF variants in the canonical transcripts of the respective genes. These variants were present in 14 genes and the list included 10 splice-site, 11 stopgain and 9 frameshift variants. Systematic annotation according to the ACMG/AMP guidelines yielded 6 variants as pathogenic and the remaining 24 variants as VUS (Fig. 2B).

Pathogenic variants were revealed in *CASQ2* (*n* = 2), *KCNQ1* (*n* = 2), *TRDN* (*n* = 1) and *PKP2* (*n* = 1) genes. Of the two variants found in the *CASQ2* gene, one was a stopgain variant, p.E236* and the other was a splicing variant c.420 + 2 T > C. Calsequestrin (CASQ2) is a calcium binding protein in the sarcoplasmic reticulum of cardiomyocytes and plays a key role in calcium homeostasis. We identified two pathogenic frameshift variations, p.W120* and p.G179Sfs*62 in the *KCNQ1* gene. Loss of function variations in this potassium channel encoding gene are associated with the disease phenotype. Furthermore, stopgain variants p.Q513* and p.R413* were noted in *TRDN* and *PKP2* genes, respectively. The Triadin (TRDN) is an important component of the calcium release unit in the sarcoplasmic reticulum of cardiomyocytes that interact with both ryanodine receptor (RYR2) as well as calsequestrin (CASQ2). Plakophilin2 (encoded by *PKP2*) is a desmosomal protein found in the intercalated discs of cardiac cells. The p.R413* variation was first identified in a Caucasian male with arrhythmogenic right ventricular cardiomyopathy (ARVC) [41]. Later on, Alcalde et al. in 2014 reported the same variant to be segregating in a Hispanic family with ARVC [42]. The summary of pathogenic pLoF variants is outlined in Table 2**.** All of these pathogenic variations were predicted to cause loss of protein function and deleterious by the CADD tool. The corresponding genes have an established loss of function mechanism for causing the disease.

**Table 2:** Pathogenic pLoF cardiac channelopathy variants as per ACMG/AMP guidelines

Gene	cDNA change	AA change	dbSNP ID	Variant type	Functional or segregation evidence	ClinVar	ACMG/AMP attributes	ACMG/AMP classification
CASQ2	c.706G > T	p.E236*	NA	stopgain	NA	NA	PVS1PM2PP3	Pathogenic (Ic)
CASQ2	c.420 + 2 T > C^$^	NA	NA	splicing	NA	NA	PVS1PM2PP3	Pathogenic (Ic)
TRDN	c.1537C > T	p.Q513*	rs757355311	stopgain	NA	Pathogenic	PVS1PM2PP3PP5	Pathogenic (Id)
PKP2	c.1237C > T	p.R413*	rs372827156	stopgain	Heterozygotes for the variation showed ARVC phenotype in a family	Pathogenic	PVS1PM2PP1PP3PP5	Pathogenic (Id)
KCNQ1	c.360del^$^	p.W120*	NA	frameshift deletion	NA	NA	PVS1PM2PP3	Pathogenic (Ic)
KCNQ1	c.524_534dup	p.G179Sfs*62	rs879255588	frameshift insertion	NA	Pathogenic/likely_pathogenic	PVS1PP5PM2PP3	Pathogenic (Id)

*AA* amino acid; *NA* not available; *ARVC* arrhythmogenic right ventricular cardiomyopathy; *$* unique variants; *stop codon

The 6 pathogenic cardiac channelopathy-associated pLoF variants were found to be present in 7 out of 1029 individuals. The variants *CASQ2*:p.E236*, *CASQ2*:c.420 + 2 T > C, *PKP2*:p.R413*, *KCNQ1*:p.W120* and *KCNQ1*:p.G179Sfs*62 were present in 1 individual each whereas the variant *TRDN*:p.Q513* was present in 2 individuals. All these individuals carry the respective variants in heterozygous state and the variants have been validated by sanger capillary sequencing. The primer details are summarised in Additional file 4. The variations in genes *CASQ2* and *TRDN* are reported to be highly penetrant and follow an autosomal recessive mode of inheritance. On the other hand, variations in genes *PKP2* and *KCNQ1* majorly follow autosomal dominant mode. Individuals with pLoF variations in these genes may be at risk of developing the respective channelopathy disorders.

The complete details of 30 pLoF variants along with their ACMG/AMP annotations have been provided in Additional file 6.

### Variants unique to the Indian population in cardiac channelopathy-associated genes

**Fig. 3:**
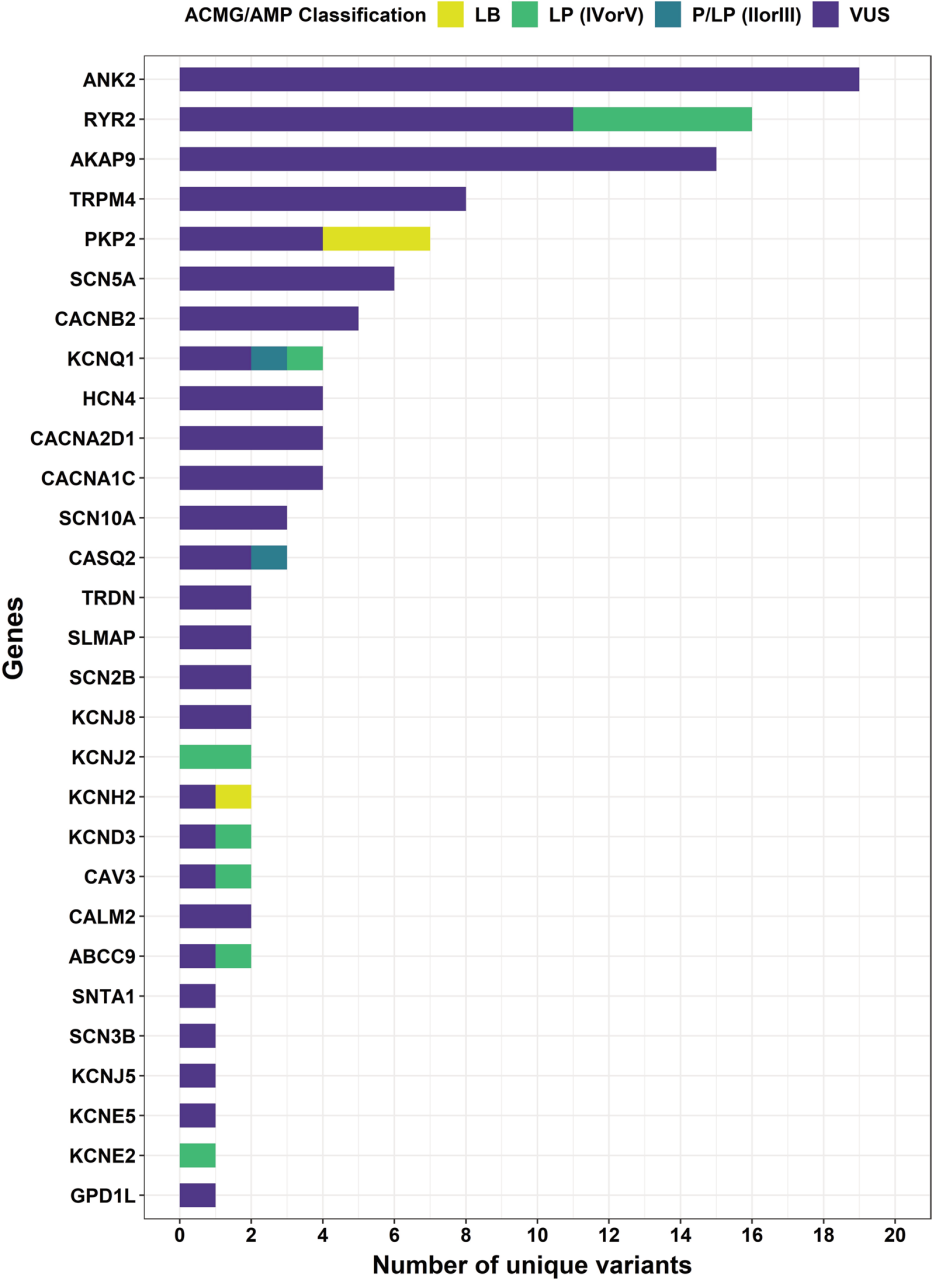
Distribution of 124 unique variations across genes with their ACMG/AMP classification. The number of variations corresponding to the genes are plotted as distinct bars. The colours in stacks correspond to the respective classification according to ACMG/AMP guidelines

Initially, we had obtained a total of 186,782 variants in 36 genes. After final filtering, the number of nonsynonymous variants reduced to 440, out of which 114 (25.9%) were unique to the IndiGenomes dataset and absent in global population datasets, publicly available databases and literature. Of these 114 unique variants, 98 (85.9%) were classified as VUS, 12 (10.5%) as low confidence, likely pathogenic, and 4 (3.5%) as likely benign. Across genes, the unique likely pathogenic variants were found in *RYR2* (*n* = 5), *KCNJ2* (*n* = 2), *CAV3, KCND3, KCNQ1, ABCC9,* and *KCNE2* (*n* = 1 each) genes. The 4 likely benign variants were found in *KCNH2* (*n* = 1) and *PKP2* (*n* = 3) genes. The complete list of nonsynonymous unique variants is mentioned in Additional file 7. Similarly, in the case of pLoF variants, we observed that 10 out of 30 variants were unique to the IndiGenomes dataset. This includes 8 VUS and 2 pathogenic variants. The pathogenic variants included *CASQ2*:c.420 + 2 T > C and *KCNQ1*:p.W120*. In total, we have discovered 124 out of 470 (26.3%) variants as unique variants in the Indian population (Fig. 3). All of these variants are yet to be identified in the channelopathy patients and functionally characterised.

The above observations highlight that there are a significant number of variants that are unique and are represented only in the Indian population compared to the rest of the world.

### Allele frequency comparison of P/LP variants across various population genome datasets

We sought to understand the significant allele frequency differences between the Indian population dataset and rest of the global population datasets. Allele frequencies of 13 ACMG/AMP classified pathogenic and high confidence likely pathogenic variants were fetched from the IndiGenomes dataset. Out of these 13 variants, 7 were nonsynonymous (*P* = 1, LP = 6) and 6 were predicted loss of function (*P* = 6).

**Fig. 4:**
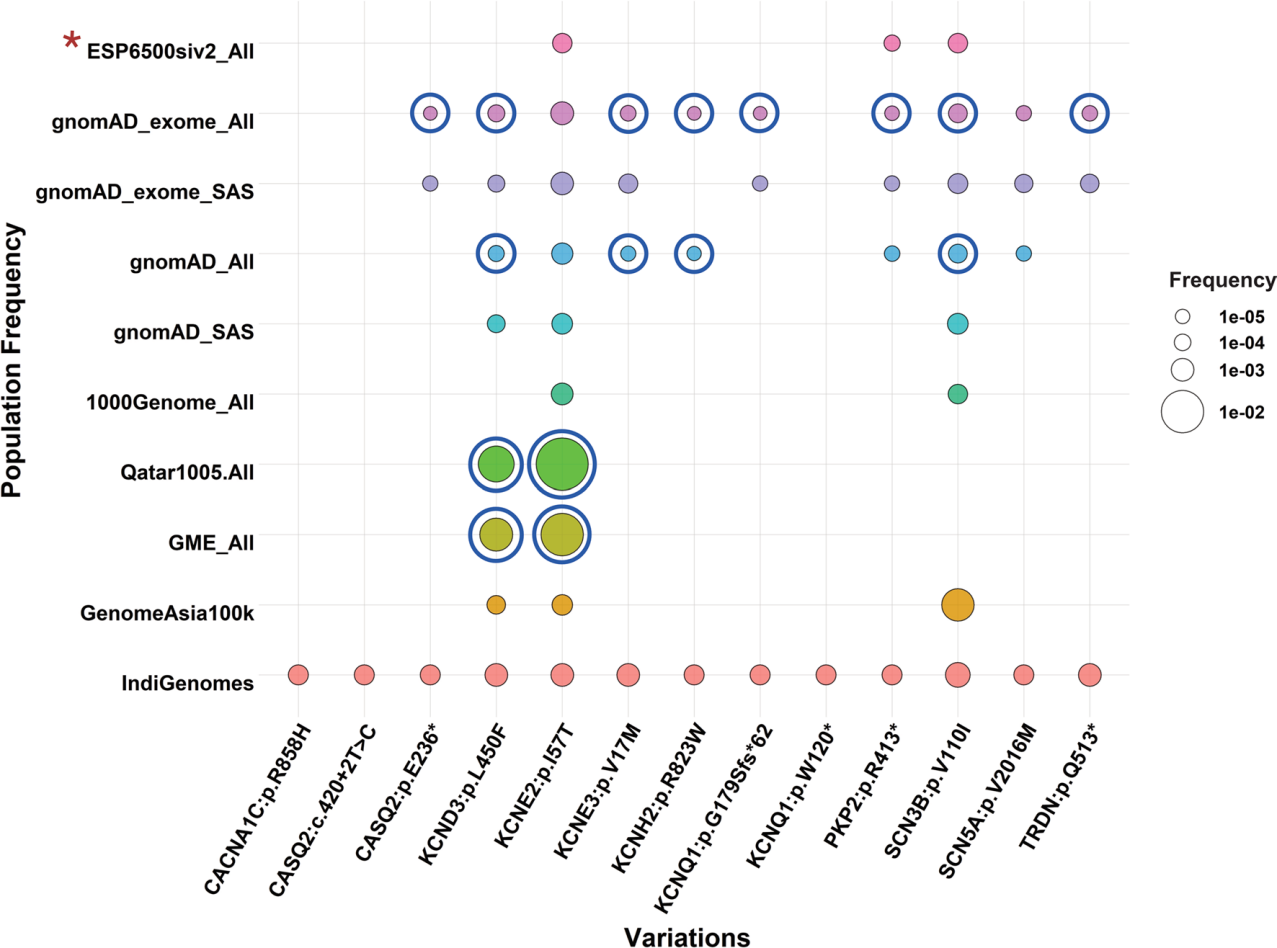
Comparison of allele frequencies of pathogenic and likely pathogenic cardiac channelopathy variants across different genomic datasets. The variant allele frequencies in different populations are plotted as solid bubbles (with filled colours). The circles outside the bubbles represent the significantly different allele frequency values (Fisher’s exact test; *p* < 0.05) using IndiGenomes dataset as a reference. Red asterisk: Fisher’s exact test was not done in case of ESP6500siv2_All dataset due to unavailability of allele numbers and allele counts

Three variations namely, *CACNA1C*:p.R858H, *CASQ2*:c.420 + 2 T > C and *KCNQ1*:p.W120* were represented only in the IndiGenomes dataset and absent in other global population datasets (Fig. 4). The remaining 10 variations were represented in the gnomad_exome_All dataset. Except for *KCNE2*:p.I57T and *SCN5A*:p.V2016M, all of them were enriched in the IndiGenomes dataset as compared to the gnomad_exome_All dataset (*p* value < 0.05, Fisher’s exact test). However, on comparing the IndiGenomes frequencies specifically with the gnomad_exome_SAS dataset, differences between allele frequencies were not significant suggesting that amongst the available global datasets, SAS dataset in gnomAD is a better representative of allele frequencies in Indian population.

Only two of the variations, i.e. *KCNE2*:p.I57T and *SCN3B*:p.V110I, were represented in the 1000 genome_All dataset. In both of the cases, there were no significant differences between the allele frequencies when compared with the IndiGenomes dataset. None of the variations was present in the 1000genome_SAS dataset.

In comparison with region-specific genomic datasets such as Qatar and GME, we found that the variations *KCND3*:p.L450F and *KCNE2*:p.I57T were enriched in the Qatar and GME datasets as compared to the IndiGenomes dataset indicating a higher genotypic prevalence of Brugada syndrome-associated risk alleles in the Middle East. Lastly, only three variations, *KCND3*:p.L450F, *KCNE2*:p.I57T and *SCN3B*:p.V110I, were represented in the GenomeAsia100k dataset. Allele frequencies for all of them did not differ significantly as compared to the IndiGenomes dataset. The allele frequencies of P/LP cardiac channelopathy variants across different genomic datasets are provided in Additional file 8.

### Intersecting pathogenic and high confidence likely pathogenic variants in an independent cardiac ion channelopathy patient cohort

We intersected the 13 ACMG/AMP classified P/LP variations obtained from the IndiGenomes dataset with the exome sequencing dataset from a patient cohort provisionally diagnosed with cardiac channelopathy disorder (*n* = 53). Consequently, we found that 3 out of the 13 variations were present in the patient cohort data.

Our analysis revealed a heterozygous individual for a pathogenic frameshift variation, *KCNQ1*:p.G179Sfs*62. The same variation was also found in a patient with provisional diagnosis of Jervell and Lange-Nielsen Syndrome in the cardiac channelopathy cohort. The variation was present in a homozygous state in the patient. Further investigation in the cardiac channelopathy cohort revealed an LQTS patient with ECG abnormality carrying a variation, *KCNH2*:p.R823W which is also identified in the IndiGenomes. In addition to these two variations, a pathogenic splicing variation, *CASQ2*:c.420 + 2 T > C was found to be overlapping between IndiGenomes and the cardiac channelopathy cohort. The variation was found to be present in a heterozygous state in both datasets. However, the *CASQ2*:c.420 + 2 T > C variation was not able to explain the complete phenotypic spectrum of the patient, which is being investigated. These findings underscore the clinical utility of our analysis of channelopathy variants in the healthy Indian population.

## Discussion

Cardiac ion channelopathies cause serious electrical disturbances in the heart that can even lead to sudden death. In addition to clinical workup and relevant family history, genetic testing has proved to be crucial to confirm the diagnosis and also for effective management. With timely diagnosis and clinical intervention, not only the fatal consequences can be prevented but the patients can lead a better quality life.

Due to advancements in genomic technologies in the past two decades, a large number of sequencing initiatives have been carried out. Particularly, population-specific genomic datasets are of great importance in distinguishing the rare variants from common variations and thus, aid in correct clinical interpretation. Our objective was to discover unique Indian variations and to gain insights into the spectrum of probable pathogenic variations in 36 genes associated with cardiac ion channelopathies and their frequencies. It is to be noted that unique variations are not specific to a set of genes but given that many of our genes are clinically actionable, it became imperative to identify unique variations. According to Adler et al.[29] and the ClinGen working group [30], 12 out of 36 genes have strong/ definitive evidence for pathogenicity and 22 genes have been classified as disputed/ limited evidence genes for inherited channelopathy disorders (Additional file 1). The remaining 2 genes (*KCNE1* and *KCNE2*) have strong evidence for acquired LQTS but are disputed/limited for inherited form of the disease. These genes have limited evidence based on the observations predominantly from the Caucasian population where cardiac channelopathy-specific studies and also a large number of exomes and genomes have been conducted. There is very little representation from other populations around the world. Since genomics is an evolving field with rapid addition of variant information almost on a daily basis which implies that although these genes have disputed evidence at present, as more evidence (functional assays, family segregation studies, large case control studies) builds up they may be upgraded in future. Despite being classified as limited/disputed evidence genes, most of them code for either ion channel subunits or scaffold proteins that are expressed in the heart and play significant roles in cardiac rhythmicity. A few variants in limited/disputed evidence genes have also been reported to alter the protein function which substantially contribute to their pathogenicity classification according to ACMG/AMP guidelines. These variants can either lead to the phenotypic defects in a standalone fashion or, taking into account the potential oligogenic nature of the disease, they can have minor effects contributing to the overall phenotype. These genes are also part of commercially available targeted gene panels for cardiac channelopathies already in practice in several parts of the world [43–46] and therefore, we aimed to identify the prevalent variations in all of the 36 genes and assess them for the Indian population.

We employed standard ACMG/AMP guidelines to classify the variants in 36 cardiac channelopathy-associated genes. This compendium of variations can serve as a go-to resource for clinicians when any of these variations are found in patients (Additional files 5 and 6). From this collection of variants, 13 were classified as pathogenic or high confidence likely pathogenic variations in the IndiGenomes dataset. These 13 variations are harboured in genes with varying degrees of evidence for pathogenicity. They were found in 19 out of the 1029 individuals in which 9 variations in definitive genes mapped to 10 out of 1029 individuals resulting in an estimated genotypic prevalence of 1:103–1:54 (0.9–1.8%) in the healthy Indian population. As an independent validation, we identified 3 out of 13 P/LP variants in a provisionally characterised cardiac disease cohort of 53 probands. In a country of 1.3 billion individuals, even a modest prevalence of 0.9–1.8% assumes a staggering proportion of public health burden. In addition, India is a culturally heterogeneous population with specific marriage patterns and the variations, as well as associated traits tend to accumulate within communities. Consequently, the founder effect is magnified which implies that unravelling the population-specific variations can accelerate disease diagnosis not only at an individual level but also at a community level. Population-based screening can help in identifying the individuals with the P/LP variations and can then be extrapolated to screen the associated communities in order to reduce the burden on public health.

By comparing the allele frequencies of IndiGenomes dataset and the resources such as 1000G, gnomAD and ExAC, we wanted to highlight the significant differences between the allele frequencies and also the utility of increasing the representation of Indian genomes in the population datasets. Interestingly, we have identified a few pathogenic variations such as *CASQ2*:p.E236*, *KCNE3*:p.V17M that were rare according to the global datasets but fairly common in the IndiGenomes dataset. Furthermore, the allele frequency differences were not significant when an ancestry-matched dataset was used (SAS) corroborating that our data is a good reflection of the SAS data. However, we highlighted that in spite of belonging to the same ancestry, there were variations such as *CACNA1C*:p.R858H, *CASQ2*:c.420 + 2 T > C, *KCNH2*:p.R823W and *KCNQ1*:p.W120* in IndiGenomes dataset that were not represented even in SAS datasets. Overall, we observed that about 26% of the investigated variants in our study were unique (absent in global population datasets, publicly available databases and published literature). Therefore, while SAS datasets are useful, we believe that as more sequences get deposited from the Indian subcontinent, the comparisons will become more reasonable and logical. This will not only aid in clinical interpretations of people residing in the country but also would be of immense value for people of same ancestry across the globe.

Intriguingly, enrichment of pathogenic and likely pathogenic variants in IndiGenomes dataset as compared to the gnomAD_All dataset, raises two possibilities (i) either the Indian population is more susceptible to channelopathy disorders or (ii) these variants are common variants with small effects and are rather disease predisposing than being actually causal. Till now, we are unaware of the actual prevalence of these disorders in India so we cannot rule out the first possibility. The second possibility could be true as the recent reports indicate the potential oligogenic inheritance of these disorders [47, 48]. In any case, these variations when found in Indian channelopathy patients should be dealt with caution. Interpretation should be made based on cumulative factors such as relevant family history, associated triggers/environmental factors and the presence of other variations explaining the disease phenotype observed in patients. This highlights the fact that the finding of only risk genotype without relevant family history, clinical examinations and other risk factors does not qualify an individual to be a patient suffering from channelopathy disorder.

Our study emphasises the need for more Indian population-specific case–control studies to establish the distribution of the described variants in the cases versus controls and large pedigrees with complete clinical and genetic workup to help assess the degree of pathogenicity of each variant. Population screening for cardiac channelopathy variations has an enormous impact on public health as it can help in the identification of individuals at risk and thus, can reflect upon the actual disease risk. This argument was very well emphasised by a recent multi-centre cohort study in which 10 arrhythmia genes were screened in > 20,000 participants without any indication for arrhythmia disorders. About 0.6% of individuals carried P/LP variants and diagnosis could be made after variant results were returned in 0.05% of the cases [49]. For the present study, we acknowledge the fact that genomic dataset of 1029 individuals is very small for a country of 1.3 billion people, nevertheless, our study only serves as the starting point in the direction to derive Indian population-specific genotypic prevalence estimates for cardiac ion channelopathy disorders. To better understand the prevalence of rare variants associated with cardiac channelopathy disorders in a country like India, one would need a larger genomic dataset. This demands an increase in Indian population-specific genome sequencing efforts. IndiGenomes dataset is one of the first whole-genome sequencing-based dataset consisting of self-declared healthy individuals from India. We envision more Indian genomes to be added in future to improve upon the accuracy of population-specific allele frequencies. Education and awareness for integrating genomics into clinical practice would serve as a stepping stone towards this effort.

## Conclusion

In conclusion, our study reveals a high prevalence of channelopathy-associated variations in the Indian population. Additionally, we found that about 26% of discovered variations were unique to the Indian population. This study underscores the importance of large-scale population genomic studies to uncover the landscape of disease-associated variations and to identify the disease burden of channelopathy disorders.

## Supplementary Information

**Additional file 1**: List of major genes associated with Cardiac Channelopathies.

**Additional file 2**: Additional Methods.

**Additional file 3**: Summary of obtained variants with MAF<0.05 after filtering.

**Additional file 4**: Primer set sequences for screening the P/LP variations.

**Additional file 5**: List of 440 nonsynonymous variations with their ACMG/AMP classification.

**Additional file 6**: List of 30 predicted loss of function variations with their ACMG/AMP classification.

**Additional file 7**: List of 114 unique nonsynonymous variations in IndiGenomes with their ACMG/AMP classification.

**Additional file 8**: Allele frequencies of pathogenic and likely pathogenic cardiac channelopathy variants across different genomic datasets.

## Data Availability

The datasets supporting the conclusion of this article are included within the article and in the Additional Material. The datasets generated and analysed during the current study have been made available for download by our group at the IndiGenomes database website IndiGenomes Resource of Population Genomes from India. https://clingen.igib.res.in/indigen/ [22].

## Abbreviations

ACMG/AMP: American College of Medical Genetics and Genomics/Association for Molecular Pathology
P/LP: Pathogenic/likely pathogenic
pLoF: Predicted loss of function
LQTS: Long QT syndrome
BrS: Brugada syndrome
CPVT: Catecholaminergic polymorphic ventricular tachycardia
SQTS: Short QT syndrome
gnomAD: Genome Aggregation Database
ESP6500: NHLBI GO Exome Sequencing Project
ExAC: Exome Aggregation Consortium
GME: Greater Middle East
VEP: Variant effect predictor
LOFTEE: Loss-of-function transcript effect estimator
GUaRDIAN: Genomics for Understanding Rare Diseases: India Alliance Network
AIIMS: All India Institute of Medical Sciences
VUS: Variant of uncertain significance
CASQ2: Calsequestrin
TRDN: Triadin
PKP2: Plakophilin2
ARVC: Arrhythmogenic right ventricular cardiomyopathy
