# Supplementary material for: 1029 genomes of self-declared healthy individuals from India reveal prevalent and clinically relevant cardiac ion channelopathy variants
Source: Hum Genomics. 2022 Aug 5;16:30. doi: 10.1186/s40246-022-00402-2 (PMC9354277; doi:10.1186/s40246-022-00402-2)
Supplement: Supplementary file 2 — Additional file 2: Additional Methods. [file 40246_2022_402_MOESM2_ESM.docx]

**Additional Methods**

**ACMG/AMP classification for Nonsynonymous variations**

Each variant was individually assessed and classified according to the ACMG/AMP guidelines. Attributes were assigned to variants based on the consensus between two curators.

For attributes related to the population frequency (PM2, BS1, BA1) the maximum of frequencies from gnomAD_all (genome and exome), 1000Genomes_all alongwith their SAS datasets and ESP6500_all was considered for the cutoff. **PM2** was assigned to any variant which had an allele frequency of 0 or less than 0.05%. For variants with allele frequencies in the range of 0.05% to 1%, no attributes were assigned. For variants with allele frequencies 1% to 5%, and greater than 5%, **BS1** and **BA1** were considered respectively. However, BA1 could not be assigned to any variations since variations having MAF>5% were already filtered out. The attribute **BS2** was not considered since channelopathies show incomplete penetrance.

For the attributes of computational prediction (PP3, BP4), predictions from SIFT, PolyPhen and CADD were considered. A variant was assigned **PP3** when two or more of the predictions were deleterious. **BP4** was assigned when two or more predictions were benign. No attributes were assigned in this category, when a variant did not meet the above criteria.

Classification of variants from the ClinVar database were considered to assign **PP5**, **BP6** attributes. The former was assigned when a variant was reported as pathogenic or likely pathogenic in Clinvar, whereas the latter was assigned when a variant was reported as benign or likely benign.

A PFam database search was done based on the Refseq IDs of the major transcripts of the corresponding gene. The attribute **PM1** was assigned to a variant, only when it was located within one of the PFam annotated functional domains of the respective transcript.

For missense variants, based on the number of variants present in ClinVar database for the respective gene, the attribute **PP2** was assigned for a variant when more than 50% of the reported pathogenic and likely pathogenic variants were missense. **BP1** was assigned when more than 50% of the reported pathogenic and likely pathogenic variants were nonsense variants.

For literature based attributes, literature search was performed using the Pubmed and Google scholar articles. Firstly, attributes PS3 or BS3 were assigned based on the evidence of functional studies in available literature. The attribute **PS3** was assigned when there was convincing evidence that a variant significantly altered the function of a protein *in-vitro* or *in-vivo*. When a variant was shown to act similar to the wildtype protein, the attribute **BS3** was assigned. No attributes were assigned in this category when a variant lacked any functional studies.

Based on the presence of the variant in cases and controls, the attribute **PS4** was assigned when a study showed that the Odds ratio was more than 5 in case-control studies.

Based on segregation data available in literature, the attribute **PP1** was assigned when the variant was found to be segregating in multiple families in one or more populations. If the variant was not segregating with the phenotype in the family, then **BS4** was considered. However, due to lack of literature evidence, BS4 could not be assigned to any variation.

The attribute **PM5** was assigned in case of novel variation, at the same amino acid residue where a different missense variation was reported to be pathogenic previously.

Due to insufficient evidence in the literature we could not assign PS2, PM3, PM6, PP4, BP5 and BP2 attributes to any of the variations. Moreover, based on the variation type, the attributes PS1, PM4, BP3 and BP7 were excluded.

**ACMG/AMP classification for predicted loss-of-function variations**

Attribute **PVS1** was assigned to variants which were annotated to be frameshift, stopgain or splice site variants and stringently follows the conditions[^1^](https://paperpile.com/c/nN3V4t/bRo0):

i) present in genes which had definitive/strong/moderate evidence for cardiac ion channelopathy in ClinGen[^2^](https://paperpile.com/c/nN3V4t/wFei) and

ii) present in genes which had loss of function as an established mode of pathogenicity according to ClinVar and

iii) variants that were located on the major transcript and

iv) variants that were predicted to have a very strong adjusted PVS1 strength according to AutoPVS1[^3^](https://paperpile.com/c/nN3V4t/t9Mw) tool. This very strong attribute was assigned only to variants which met the above criteria.

The attributes PM2, BS1, PP5, BP6, PS3, BS3, PS4, PP1, BS4 were considered as described above. For PP3, BP4 attributes, FATHMM, MutationTaster and CADD were considered. Moreover, PM1 was not considered as functional domain information was included in the PVS1 attribute. All the remaining attributes were not considered either due to insufficient literature evidence or due to type of variation.

**References:**

1. [Abou Tayoun AN, Pesaran T, DiStefano MT, et al. Recommendations for interpreting the loss of function PVS1 ACMG/AMP variant criterion. *Hum Mutat*. 2018;39(11):1517-1524.](http://paperpile.com/b/nN3V4t/bRo0)

2. [Strande NT, Riggs ER, Buchanan AH, et al. Evaluating the Clinical Validity of Gene-Disease Associations: An Evidence-Based Framework Developed by the Clinical Genome Resource. *Am J Hum Genet*. 2017;100(6):895-906.](http://paperpile.com/b/nN3V4t/wFei)

3. [Xiang J, Peng J, Baxter S, Peng Z. AutoPVS1: An automatic classification tool for PVS1 interpretation of null variants. *Hum Mutat*. 2020;41(9):1488-1498.](http://paperpile.com/b/nN3V4t/t9Mw)
